# New Trends and Future Perspectives in the Diagnosis of Urothelial Carcinoma: A Comprehensive Review of the Literature

**DOI:** 10.3390/medicina61010071

**Published:** 2025-01-03

**Authors:** Mourmouris Panagiotis, Georgopoulos Ioannis, Klapsis Vasilleios, Pisiotis Nikolaos, Salmas Ioannis, Charamoglis Sotirios

**Affiliations:** Urology Department, Metropolitan Hospital, Neo Faliro, 18547 Piraeus, Greece; johngeorgop@yahoo.gr (G.I.); billklapsis@hotmail.com (K.V.); dr.pissiotis@gmail.com (P.N.); giannis.salmas@yahoo.gr (S.I.); haramogliss@gmail.com (C.S.)

**Keywords:** urothelial carcinoma, diagnosis, bladder cancer, upper urinary tract cancer, imaging, endoscopy, biomarkers, cytology

## Abstract

Despite the high incidence of bladder cancer (it represents the 7th most common cancer in males), EAU guidelines do not recommend any technique for screening and prevention, whereas the main diagnostic tools remain computed tomography urography (CTU), cytology, and cystoscopy. Unfortunately, these gold-standard modalities are mainly characterized by low sensitivity and accuracy. To minimize the limitations and increase the detection rates of urothelial cancer, several technologies have been developed. In our review, we summarize all the available data concerning the contemporary technological novelties that include imaging, cytology, and endoscopic techniques in pursuit of finding the optimal combination of diagnostic tools that can provide better detection accuracy.

## 1. Introduction

The urinary tract extends from the renal calyces to the urethra so urothelial cancer may occur in any of these anatomical landmarks. The most prevalent cancer of the urothelium worldwide is urinary bladder cancer (BC), with an incidence rate (per 100.000 person/years) of 9.5 in males and 2.4 in females [1], whereas the rates for upper urinary tract (UUTUC) and urethral (UC) malignant tumors are 2.0 [2] and 0.004 [3], respectively. In BC and UUTUC, the incidence rates are higher in men, whereas in UC, the opposite is true. Despite the relatively high incidence of bladder cancer (it represents the seventh most common cancer in males), EAU guidelines do not recommend any technique for screening and prevention, whereas the main diagnostic tools remain computed tomography urography (CTU), cytology, and cystoscopy [4]. Unfortunately, all of them are characterized by several drawbacks. CTU sensitivity decreases as the lesion size diminishes, with rates of 89% for lesions <5 mm and nearly 40% if the size of the tumor is <3 mm accompanied by possible adverse effects of radiation and contrast material [5]. Cytology is characterized by its low sensitivity for low-grade tumors, whereas cystoscopy is characterized by its invasive nature and its inherited limitations of differentiating flat tumors and standard white light [6]. Considering these difficulties, we review the literature for the most recent advances in the diagnosis of this important medical entity with a goal of reinforcing our clinical diagnostic tools.

## 2. Material and Methods

**Figure 1:**
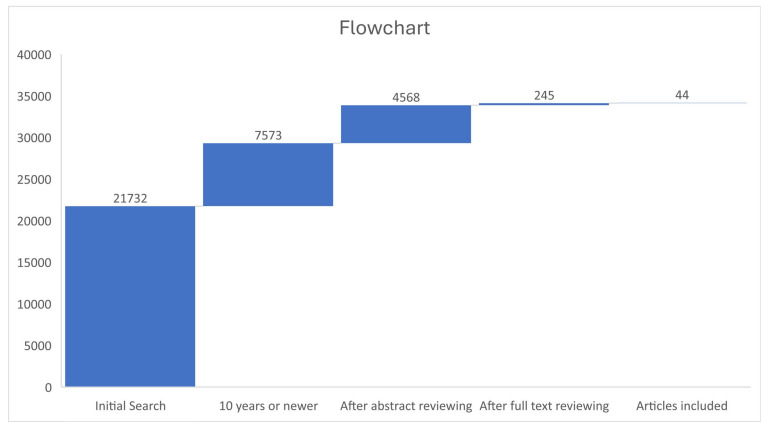
Flowchart of included studies.

Our search strategy was as follows: “(Urothelial Cancer) OR (urothelial carcinoma) OR (Transitional cell carcinoma) OR (Urine cytology) OR (Intravenous pyelography) OR (urologic oncology) OR (diagnosis) OR (magnetic urography) OR (cystoscopy)”. The search was performed in PubMed/Medline, Scopus and Web of Science and was limited to papers reporting at least an abstract and only in English language. Since the scope of our research is to report contemporary trends, the search was performed between the years 2010 to 2024. We included original research, systematic reviews, meta-analyses, and case series only if they included novel techniques. The evaluation of the papers was performed by two researchers, P.M. and I.G., separately. We excluded simple reviews, case reports, and papers that reported only the gold-standard diagnostic entities. The flowchart of our search is depicted in Figure 1.

## 3. Imaging Techniques

### Computed Tomography Urography

CTU is the gold standard technique that is proposed by most of the guidelines worldwide for the diagnostic management of hematuria and urothelial cancer. There have been efforts to maximize its efficacy with multiple novel variations. These variations refer to the time of contrast administration (singe bolus, split bolus, etc.) [7,8], image reconstruction, and post-image processing, including 3D reconstruction [9,10] or the energy used in its protocols (dual energy-DECT) [11]. These techniques have their advantages and disadvantages; unfortunately, none of these efforts can overcome the basic limitations of the technique, which is the radiation exposure and the inability to utilize it in patients suffering from kidney failure. Therefore, it is important to stress the importance of artificial intelligence (AI), which is the most novel addition to our armamentarium. Except from its utilization on segmentation of bladder lesions and radiomics analysis, which can significantly aid in distinguishing between cancer and non-cancerous lesions [12], there is another promising field on bladder cancer staging: invasion of the bladder muscle. A new deep-learning model that used CTU images demonstrated a promising accuracy (ROC curve (Az) from 0.89 to 0.97) in the determination of muscle invasion of patients with bladder tumors [13]. The use of AI as an imaging diagnostic tool, despite its rapid development, is still in its infancy with several drawbacks, and for the time being, it should be considered as an auxiliary method that could potentially increase the accuracy of the traditional methods.

## 4. Magnetic Resonance Imaging Urography

Magnetic Resonance has been used for many years in the diagnosis mainly of kidney tumors, axillary to CT. The new protocols of MRU have been added to our everyday clinical practice as an alternative to the CTU, especially when there is a contraindication for the latter. Nevertheless, the MRU might be considered a standard tool that can provide important information about urothelial cancer as long it includes acquisition dedicated to the urinary collective system with at least TSE/FSE T2 weighted, T1/T2 anatomical fast, and DWI imaging [14]. Furthermore, the most emerging diagnostic application of the MRI is the multiparametric MRI (mpMRI) mainly for the evaluation of the muscle invasiveness of bladder cancer. The clinical importance of mpMRI relies on the VIRADS score (Vesical Imaging Reporting and Data System) that extends from 1 to 5, with a score of 1 representing a very low probability of muscle invasion and 5 being very likely cancer above T2 [15]. The sensitivity and specificity for addressing invasion status are reported to be as high as 91–95% and 68–79%, respectively, with an inter-reader agreement of 0.79 [16] and a receiver operating curve (AUC) of 0.92 [17]. These results are validated with a recent meta-analysis of 20 studies that concluded that the diagnostic performance of VI-RADS of the staging of bladder tumors before resection is very promising and could be implemented in everyday clinical practice [18].

## 5. Urine Cytology

Despite the fact that conventional urinary cytology is still considered as gold standard for the detection of urothelial cancer, we must stress the low sensitivity and specificity of this method can be as low as 20% and 74%, respectively [19]. Considering these disappointing results there have been authors that reported in the literature methods that were pointing on increasing the accuracy of cytology. One of these relatively novel techniques is the multicolor fluorescence in situ hybridization assay (FISH) technique that aims to detect abnormal nuclei and cells with aneuploidy and gene deletions, basically in chromosomes 3,17 and 17. The results were extremely promising, with a remarkable increase of sensitivity to 59–92% and specificity to 66–90% [20,21]. At the same pace, another contemporary staining technique, Cell Detect, has been proposed by Shang et al. The authors followed more than 250 patients, with 2 cytologists reviewing the produced slides and reporting an increased intra-observer accuracy due to easy identification of malignant cells. This study reports a 51% sensitivity for low-grade tumors and 92% for high-grade tumors of the standard cytology, whereas the sensitivity and specificity of Cell Detect were found to be 82% and 88% significantly increased when compared to conventional cytology [21]. Again, focusing on real-world data, a blinded prospective study confirmed the high accuracy of this method irrespective of tumor grade [22]. The most recent data about the real-world value of these techniques are reported from the meta-analysis of 13 papers and more than 1000 patients [23]. The authors reported an increased specificity and sensitivity with an excellent AUC of 0.91. However, the basic limitation of all cytology methods remains the inter- and intra-observer reproducibility despite the universal Paris grading system [21]. Most recently, artificial intelligence has been applied in order to lower or eradicate this drawback. The development of an AI-assisted digital workflow managed to improve the sensitivity of microscopy from 87.2% to 92% and negative predictive value (NPV) from 94% to 96%, but most importantly, it demonstrated a near-perfect agreement (k = 0.862) [24]. Artificial Intelligence, despite its limited implementation in the diagnosis of urothelial cancer resulting in a low level of evidence, is possibly the most promising tool for the future of observer-depending methods.

## 6. Biomarkers

The limitations of urinary cytology turned the attention of researchers toward more advanced molecular diagnostic tools. The research was expanded with many different markers being currently under evaluation. Table 1 summarizes the most cited novel markers proposed for the diagnosis of UC [25,26,27,28,29,30]. From these, the most promising are considered the NMP22 and the bladder tumor antigen (BTA), both reinforced with considerable evidence and FDA approval. The first one is a non-chromatin protein with a sensitivity of 59%, specificity of 89%, and area under the curve (AUC) of 0.83, which is considered excellent for a biomarker, according to a recent meta-analysis [31]. Moreover, BTA detects human complement factor H-related protein and performs relatively well with 69% and 77% sensitivity and specificity, respectively, with increasing accuracy related to cancer grade [32]. Other novel biomarkers include CK20, which exhibits a sensitivity and specificity of 83% and 77%, respectively [25], and the survival of the inhibitor of apoptosis protein family (IAP). Despite the fact that the latter can provide a specificity reaching as high as 100%, there is currently no standardized method to detect it in urine samples [30]. It must be stressed that biomarkers can be used as a follow-up and not only as an initial diagnostic tool to decrease the necessity of most invasive procedures. This was the goal of a recent study that included 629 patients and utilized an adxbladder biomarker that demonstrated a sensitivity of 67% and a negative predictive value (NPV) of 99% for the detection of high-grade (HG)/CIS bladder cancer. The probability of this kind of recurrence in adxbladder-positive patients was 5% and in negative 0.85% [33].

Recently, authors have published studies utilizing gene sequencing methods, which is the most contemporary tool in biomarker-based diagnosis of urothelial cancer. In one prospective and multicenter study, authors developed four diagnostic gene expression signatures, with one of those (GS_D2) managing to reach a sensitivity (SN) of 68% for low-grade tumors and 89% for high-grade tumors with an impressive AUC of 0.918. According to the authors, their gene expression test demonstrates a diagnostic accuracy equal to or even superior to the combination of cystoscopy plus cytology [34].

## 7. Endoscopy

The mainstay of diagnosis of urinary tract cancer is endoscopy (cystoscopy-ureteroscopy). It must be performed in every case presenting with hematuria (cystoscopy) and can be decisive in exploring pathologies of the upper urinary tract when imaging is not conclusive [4]. Nevertheless, this important tool has its own drawbacks, which can lead to misdiagnosis, which, in the case of urothelial cancer, could be fatal. In pursuit of increasing endoscopy accuracy, different technologies have been developed. One of these technologies is photodynamic diagnosis (PDD) which utilizes blue light along with a preoperative intravesical instillation of a fluorophore. It depends on the accumulation of the photosensitizing agent (currently hexylamino levulinate (HAL), leading to absorption from neoplastic cells and emission of red light when exposed to blue light. Since the introduction of the technique back in the 90s [35], it has evolved significantly with many empowered studies reinforcing its importance since then.

The data for these studies confirm some important conclusions. First of all, the direct impact of PDD in clinical practice with a grounding 25% of bladder cancer patients would have been missed if only white light was utilized, whereas 14% of patients have altered their management decisions according to the findings of the PPD cystoscopy [36]. Recently, the same group published the expansion of the technique to flexible cystoscopy, which can aid in cancer detection in an office-based setting [37]. The advantages of this technique can also be expanded to the upper urinary tract. Kata et al., included in one of the largest series so far, 106 renal units, and proved that PDD for the diagnosis of upper urinary tract urothelial cancer had increased sensitivity (96%) compared to white light (54%) with this result reported as statistically significant (*p* < 0.0001).

Second, significantly, a novel imaging method is narrow-band imaging (NBI). Its mechanism of action depends on revealing the neovascularization of bladder cancer after filtering white light to rule out the red spectrum. One of the largest meta-analyses conducted to compare white light and NBI provided interesting outcomes. This relatively novel technology improved approximately 10% the detection rate of cystoscopy with this improvement to be more profound in the detection of CIS whilst providing a significant reduction in bladder cancer recurrence rates [38]. The same results are reported from a recent systematic review and meta-analysis of RCTs, with a lower risk of disease recurrence of NBI compared to white light time (hazard ratio 0.63, 95% CI 0.45 to 0.89; I2 = 53%;) despite the fact that none of the included studies studied disease progression as a time to event outcome [39]. On the other hand, some recent large RCTs do not reinforce the above optimism, failing to prove any statistically significant overall recurrence reduction (in one year), although a trend for this reduction has been observed [40,41]. Of course, it must be stressed that except from the controversy in clinical efficacy, there are also the drawbacks of cost and availability of this equipment in clinical settings worldwide, but this is beyond the scope of this review.

Except for the basic novel technologies already mentioned, there are also many others that have been developed and are awaiting confirmation from scientific studies. These include the SPIES endoscopic imaging platform (Karl-Storz^®^, Tutllingen, Germany) that implements software-based modes that change the visible spectrum of the device in order to improve contrast with better outcomes compared to white light according to a recent study [42]. Furthermore, optical coherence tomography (OCT) has been successfully paired with endoscopic platforms. This technology can aid in the early detection of the bladder since it has the potential to discriminate between superficial and invasive tumors (T1 or above) with an excellent NPV (89%) [43]. Finally, another promising innovative tool is confocal laser endomicroscopy (CLE), which has been utilized recently mainly for upper tract urothelial cancer, proving its feasibility [44]. Of course, it must be stressed that this advanced instrumentation has certain drawbacks, and despite the fact that they have been tested in clinical practice, some of them, for different reasons, have not gained a role in the initial diagnosis of urinary cancer. Most robust data from well-organized studies are needed to clarify the significance, if any, of these technologies in the diagnostic workup of patients with hematuria.

## 8. Conclusions

Urinary tract cancer represents a challenging field, especially for the diagnosis and staging. The gold standard techniques are missing a significant rate of patients since their specificity and sensitivity are limited. There is continuous research on novel tools that could potentially overcome these hurdles and provide better outcomes on diagnosis and, ultimately, management of this life-threatening tumor. These tools alone or in combination with artificial intelligence could come to significantly alter the current diagnostic workup for urinary cancer, but, of course, further research in the field of AI and radio genomics is needed.

## Figures and Tables

**Table 1:** Characteristics of basic biomarkers.

Name	Study	No of Patients	Family	Results	Advantages	Disadvantages
NMP 22 [31]	SR and MA	32,000	Non-chromatic proteins	SN 59% SP 89%	FDA approval	Low accuracy for low-grade tumors
BTA [32]	SR	7144	Detects Human complement factor H-related protein	Sn 69% SP 77%	FDA approval	Low accuracy for low-grade tumors
Cytokeratin 20 (CK20) [25]	Prospective	198	Keratin containing intermediate filaments	SN 70–77% SP 81–83%	Detection of cancer when atypical urine cytology is present	No standardized detection modality
Survinin [30]	Prospective	66	Inhibitor of apoptosis protein (IAP)	SN 35–83% SP 93–100%	Better in high-grade cancer	No standardized detection modality
BLCA1/4 [26]	Prospective	53	Nuclear Matrix protein group	SN 80–97% SP 87–100%	No correlation with grade	No clear cut-off No standardized test
Cyfra 21–1 [27]	Prospective	125	Proteolytic region of CK19	SN 82% SP 80%	Established cut-off value	Many false positive results
Apolipoproteins [28]	Prospective	451	Lipid binding proteins	SN 78–86% SP 92–100%	Good SN and SP, even for LG tumors	Need to be combined for high SN and SP
SNCG [29]	Prospective	250	Synuclein	SN 68% SP 97%	Better in early-stage tumors	High false positive results

## Data Availability

No new data were created or analyzed in this study. Data sharing is not applicable to this article.
